# Lower leukotriene C_4_ levels in bronchoalveolar lavage fluid of asthmatic subjects after 2.5 years of inhaled corticosteroid therapy

**DOI:** 10.1155/S0962935195000688

**Published:** 1995-11

**Authors:** Y. Oosterhoff, S. E. Overbeek, R. Douma, J. A. Noordhoek, D. S. Postma, H. C. Hoogsteden, F. J. Zijlstra

**Affiliations:** 1 Department of Pulmonology University Hospital Groningen Erasmus University Rotterdam The Netherlands; 2 Department of Dijkzigt University Hospital Rotterdam Erasmus University Rotterdam The Netherlands; 3 Department of Pharmacology Erasmus University Rotterdam The Netherlands

## Abstract

Long-term treatment with inhaled corticosteroids has been shown to result in improvement of symptoms and lung function in subjects with asthma. Arachidonic acid (AA) metabolites are thought to play a role in the pathophysiology of asthma. It was assessed whether differences could be found in bronchoalveolar lavage (BAL) AA metabolite levels between subjects with asthma who were treated for 2.5 years with inhaled bronchodilators alone or in combination with inhaled corticosteroids. Prostaglandin (PG)D_2_, PGF_2α_, 6-keto-PGF_1α_, thromboxane B_2_, leukotriene (LT)C_4_ and LTB_4_ levels and cell numbers were assessed in BAL fluid from 22 non-smoking asthmatic subjects. They were participating in a randomized, double-blind multicentre drug trial over a period of 2.5 years. Results of the group treated with inhaled corticosteroids (CS^+^: beclomethasone 200 μg four times daily) were compared with the other group (CS^−^) which was treated with either ipratropium bromide (40 μg four times daily) or placebo. BAL LTC_4_ levels of asthmatic subjects were significantly lower after 2.5 years inhaled corticosteroid therapy (CS^+^, 9(1–17) pg/ml *vs*. CS^−^, 16(6-53) pg/ml; p = 0.01). The same trend was observed for the PGD_2_ levels. The results suggest that inhaled corticosteroids may exert their beneficial effect on lung function via a mechanism in which inhibition of LTC_4_ synthesis in the airways is involved.


Mediators of Inflammation 4, 426–430 (1995)

### Lower leukotriene $C_4$ levels in bronchoalveolar lavage fluid of asthmatic subjects after 2.5 years of inhaled corticosteroid therapy

Y. Oosterhoff,[1,CA] S. E. Overbeek,[2] R. Douma,[1] J. A. Noordhoek,[1] D. S. Postma,[1] H. C. Hoogsteden[2] and F. J. Zijlstra[3]

Departments of [1]Pulmonology University Hospital Groningen, [2]Dijkzigt University Hospital Rotterdam and [3]Department of Pharmacology, Erasmus University Rotterdam, the Netherlands

[CA]Corresponding Author

**LONG-TERM treatment with inhaled corticosteroids has been shown to result in improvement of symptoms and lung function in subjects with asthma. Arachidonic acid (AA) metabolites are thought to play a role in the pathophysiology of asthma. It was assessed whether differences could be found in bronchoalveolar lavage (BAL) AA metabolite levels between subjects with asthma who were treated for 2.5 years with inhaled bronchodilators alone or in combination with inhaled corticosteroids. Prostaglandin (PG)$D_2$, $PGF_{2\alpha}$, 6-keto-$PGF_{1\alpha}$, thromboxane $B_2$, leukotriene (LT)$C_4$ and $LTB_4$ levels and cell numbers were assessed in BAL fluid from 22 non-smoking asthmatic subjects. They were participating in a randomized, double-blind multicentre drug trial over a period of 2.5 years. Results of the group treated with inhaled corticosteroids ($CS^+$: beclomethasone 200 µg four times daily) were compared with the other group ($CS^-$) which was treated with either ipratropium bromide (40 µg four times daily) or placebo. BAL $LTC_4$ levels of asthmatic subjects were significantly lower after 2.5 years inhaled corticosteroid therapy ($CS^+$, 9(1–17) pg/ml *vs.* $CS^-$, 16(6–53) pg/ml; $p = 0.01$). The same trend was observed for the $PGD_2$ levels. The results suggest that inhaled corticosteroids may exert their beneficial effect on lung function via a mechanism in which inhibition of $LTC_4$ synthesis in the airways is involved.**

**Key words:** Arachidonic acid metabolites, Asthma, Bronchoalveolar lavage, Corticosteroids, Leukotriene $C_4$, Prostaglandin $D_2$

### Introduction

Recent long-term studies on the prognosis and morbidity of obstructive airways disease have shown that addition of inhaled corticosteroids to maintenance treatment with a $\beta_2$-agonist in hyper-responsive patients with obstructive airways disease leads to a significant reduction of respiratory symptoms, exacerbation rates, airway obstruction and airway hyper-responsiveness.[1,2] These findings are thought to be a consequence of suppression of inflammatory processes in the airways.

No single cell type or mediator in the inflammatory processes in the airways is responsible for the clinical events in asthma. Nevertheless, there is now substantial evidence that arachidonic acid (AA) metabolites play an important role in the pathophysiology of the disease. They are potent airway constrictors, increase mucus secretion, play a role in chemotaxis and may enhance airway responsiveness, one of the characteristics of the disease.[3] A role for AA metabolites in the modulation of severity of asthma is suggested by the presence of higher levels in BAL fluid as compared to those in healthy subjects, increasing even more after allergen provocation.[4,5]

One potential mode of action of inhaled corticosteroids involves the modulation of arachidonic acid (AA) metabolite levels in the airways.[6] They may decrease AA metabolite synthesis by their inhibitory effect on phospholipase $A_2$ or by inhibition of synthesis of cytokines that stimulate AA metabolite release. In order to assess the role of AA metabolites in the effects of long-term treatment of asthmatic subjects with corticosteroids, AA metabolite levels in bronchoalveolar lavage (BAL) fluid were assessed in a subgroup of non-smoking asthmatic patients who participated in a randomized, double-blind multicentre drug trial for 2.5 years.

© 1995 Rapid Science Publishers

### Materials and Methods

*Patient selection:* Before entering the multicentre drug intervention trial, patients were selected based on: (1) Baseline $FEV_1$ larger than 1.2 l (ranging between 4.5 and 1.6 residual standard deviations below the predicted value), or an $FEV_1$/inspiratory vital capacity (IVC) ratio lower than 1.64 RSD below the predicted value, provided that total lung capacity was higher than 1.64 RSD below the predicted level;[1] (2) airway hyper-responsiveness to histamine, defined as the provocative concentration of histamine that caused 20% decrease in $FEV_1$ ($PC_{20}$) $\leqslant$ 8 mg/ml; and (3) exclusion of pregnant women, patients with a history of occupational asthma or other serious diseases, patients who used oral corticosteroids, β-blockers, nitrates, or anticoagulants, and patients who continuously used antibiotics.

At enrolment, the subjects were assigned to one of three double-blind regimens using identical metered dose inhalers: all patients received an inhaled $\beta_2$-agonist (terbutaline 500 µg) combined with either an inhaled corticosteroid (beclomethasone dipropionate 200 µg), an inhaled anticholinergic (ipratropium bromide 40 µg) or an inhaled placebo. All medication was taken four times daily.

*Study design:* AA metabolites and cell numbers were analysed in BAL fluid obtained from outpatients from two university pulmonary departments (Groningen and Rotterdam), 2.5 years after participation in the above-indicated randomized, double-blind multicentre drug intervention trial.[1] At the time of selection of patients for this study and bronchoscopy the patients were still receiving their trial medication. Therefore, the investigators were blind to the treatment that had been received in the preceding 30 months. Analysis was performed only in BAL fluid obtained from 22 non-smoking patients with asthma, diagnosed by criteria from a standardized history of respiratory symptoms[1] and with a reversibility of airways obstruction > 9% of $FEV_1$ % predicted at entry.

In accordance with findings in all participants of the trial,[1] no significant differences in $FEV_1$ values, reversibility of airway obstruction and airway responsiveness to histamine obtained at the last visit preceding the bronchoscopy procedure were found between asthmatic patients assigned to the placebo and anticholinergic groups. Therefore, differences in BAL parameters in this study were investigated between the groups without (i.e. placebo + anticholinergic therapy) and with inhaled corticosteroid therapy.

Correlations between BAL data and lung function parameters obtained 1 week preceding the bronchoscopy procedure were investigated. The study protocol was approved by the medical ethics committees of the participating hospitals. All patients gave written informed consent.

*Pulmonary function and inhalation provocation tests:* $FEV_1$ was performed on water-sealed spirometers according to standardized guidelines,[7] before and 20 min after four single inhalations of 250 µg of terbutaline administered through a 750-ml spacer device (Nebuhaler). Histamine provocation tests were performed using a 2 min tidal breathing method, as described previously.[1] Measurements were made only during clinical stable periods, and not within 4 weeks after the termination of a course of prednisolone. All pulmonary medications were discontinued 8 h before each test.

*Bronchoalveolar lavage:* Fibre optic bronchoscopy (Olympus B1 IT10, Tokyo, Japan) was undertaken according to guidelines of the American Thoracic Society.[8] Premedication consisted of intramuscular injection of 0.5 mg atropine and inhalation of 500 µg terbutaline 30 min before the procedure. Lidocaine 4% was administered into the upper airways and bronchial tree. Bronchoalveolar lavage was performed with 1 × 30 ml (pool 1) and 4 × 50 ml (pool 2) sterile phosphate-buffered saline (PBS) at 37°C with the bronchoscope wedged in the lateral segment of the right middle lobe. After recovery by gentle suction (−40 cm $H_2O$), the BAL fluid was collected in a siliconized specimen trap placed on melting ice.

Immediately after collection of the BAL fluid the laboratory procedures were carried out. The BAL fluid was centrifuged at 400 × **g** at 4°C for 5 min. BAL supernatant was separated from the cell pellet. The cell pellets were washed in PBS supplemented with 0.5% heat-inactivated bovine serum albumin (BSA). Total leukocyte numbers in BAL cell suspensions were counted in a Coulter Counter and viability was assessed by cellular exclusion of trypan blue. Cell differentials were done on May–Grünwald–Giemsa stained cytocentrifuge preparations. At least 500 cells were counted.

*AA metabolite determination:* Immediately after the BAL procedure, 20 ml of BAL supernatant from pool 2 was processed on C18 SepPak cartridges (Millipore, Bedford, USA) as described previously,[9] eluated with 2.5 ml methanol and stored at −80°C until analysis. Samples of 200 µl BAL eluted fluid were pipetted into polypropylene tubes and dried with a Savant sample con-

**Table 1.** Patient characteristics and lung function parameters at entry in the study, according to treatment group

| Characteristic | $CS^-$ | $CS^+$ | p |
|---|---|---|---|
| Number | 13 | 9 | |
| Gender, M/F | 11/2 | 5/4 | N.S. |
| Age, years | 36 (23–55) | 43 (31–60) | N.S. |
| Diagnosis, asthma/ asthmatic bronchitis | 10/3 | 6/3 | N.S. |
| Blood eosinophils ($\times 10^6$/l) | 200 (79–631) | 251 (18–501) | N.S. |
| Atopy* | 13+ | 7+/2– | N.S. |
| Total IgE (IU/ml) | 120 (18–1000) | 110 (4–1000) | N.S. |
| $FEV_1$ % pred. | 62 (38–90) | 60 (46–84) | N.S. |
| $FEV_1$/VC | 55 (38–68) | 55 (43–65) | N.S. |
| Reversibility ($FEV_1$ % pred.) | 16.6 (9–31.2) | 16.4 (9–27.3) | N.S. |
| $PC_{20}$ histamine (mg/ml) | 0.14 (0.01–0.79) | 0.16 (0.03–3.2) | N.S. |

Medians with range.
$CS^-$ and $CS^+$ = asthma groups treated without and with inhaled corticosteroids, respectively. *Atopy as determined by positive results of intracutaneous tests against house dust mite or two other tested common aeroallergens.

**Table 2.** Lung function parameters at the last visit just before bronchoscopy, according to treatment group

| Parameter | $CS^-$ | $CS^+$ | p |
|---|---|---|---|
| Blood eosinophils ($\times 10^6$/l) | 152 (59–616) | 130 (22–309) | N.S. |
| $FEV_1$ % pred. | 56 (31–87) | 88 (56–99) | 0.002 |
| $FEV_1$/VC | 51.6 (28.9–68.4) | 60.6 (53.6–75.4) | 0.01 |
| Reversibility ($FEV_1$ % pred.) | 19.0 (–0.6–36.9) | 8.5 (–3.5–19.9) | 0.01 |
| $PC_{20}$ histamine (mg/ml) | 0.06 (0.02–0.87) | 1.46 (0.1–14.4) | 0.001 |

Medians with range. $CS^-$ and $CS^+$ = asthma groups treated without and with inhaled corticosteroids, respectively.

centrator. After dissolving in 300 µl assay buffer, levels of thromboxane $B_2$ ($TxB_2$), and leukotriene $B_4$ ($LTB_4$) were determined by means of a [$^3$H]-RIA with antisera from Advanced Magnetics Inc. (Cambridge, MA) and [$^3$H]-labelled compounds from Amersham International (Buckinghamshire, UK). Levels of prostaglandins (PG) $D_2$ and $PGF_{2\alpha}$ were determined with commercially available [$^3$H]-kits (Amersham, UK) and 6-k$PGF_{1\alpha}$ with a [$^{125}$I]-RIA kit (Du Pont de Nemours, Dreieich, Germany), according to the manufacturer's instructions. Leukotriene $C_4/D_4/E_4$ ($LTC_4$) was measured at room temperature in a microtitre enzyme immunoassay according to the manufacturer's protocol (Biotrak, Amersham, UK). The cross-reactivity of the $LTC_4$ antibody with $LTD_4$ was 100% and with $LTE_4$, 30%. Cross-reactivities for the other assays to related compounds were negligible or less than 2% at B/Bo 50%.

*Data analysis:* Values are presented as medians with ranges. Spirometry data were analysed with Student's *t*-test. Bronchoalveolar lavage data were not normally distributed and therefore analysed with the Mann–Whitney *U* test. Correlations were made using Spearman's rank correlation tests. All analyses were performed with the SPSS/PC$^+$ V 4.01 software package (SPSS Inc., Chicago, IL). Values of $p < 0.05$ were considered statistically significant.

### Results

*Subjects:* Patient characteristics and lung function parameters at entry in the study in the groups with ($CS^+$, $n = 9$) and without ($CS^-$, $n = 13$) corticosteroid treatment are listed in Table 1. Comparing group data at entry in the trial retrospectively, no significant differences in patient characteristics and lung function parameters were found between the groups. In contrast, after 2.5 years of double-blind, randomized treatment, a significant improvement in $FEV_1$, reduction in reversibility of airway obstruction and reduction in airway responsiveness to $PC_{20}$ histamine were found in the $CS^+$ as compared to the $CS^-$ group (Table 2).

*BAL cell numbers and levels of AA metabolites:* The percentage recovery of BAL fluid was significantly higher in the $CS^+$ than in the $CS^-$ group ($p = 0.01$). There were no significant differences in median total or differential cell numbers/ml BAL fluid between the groups (Table 3). The median $LTC_4$ level in the $CS^+$ group was significantly lower than the level in the $CS^-$ group ($p = 0.01$), while the median $PGD_2$ level showed the same trend (Table 4). The levels of the other investigated AA metabolites were not significantly different between the $CS^+$ and $CS^-$ groups.

**Table 3.** Effect of inhaled corticosteroids on BAL fluid volume and cell numbers (pool 2)

| Parameter | $CS^-$ | $CS^+$ | *p* |
|---|---|---|---|
| Recovery % | 34 (10-68) | 63 (33-72) | 0.01 |
| Total leukocytes × $10^3$/ml | 85 (14-212) | 123 (26-431) | N.S. |
| Alveolar macrophages | 79 (9-180) | 99 (21-349) | N.S. |
| Lymphocytes | 3 (0-15) | 7 (0-78) | N.S. |
| Neutrophils | 1 (0-19) | 2 (0-7) | N.S. |
| Eosinophils | 1 (0-9) | 0 (0-4) | N.S. |

Medians with range. $CS^-$ and $CS^+$ = asthma groups treated without and with inhaled corticosteroids, respectively.

**Table 4.** BAL AA metabolite levels (pg/ml)

| Metabolite | $CS^-$ | $CS^+$ | *p* |
|---|---|---|---|
| $PGD_2$ | 77 (15-200) | 28 (17-138) | 0.12 |
| $PGF_{2\alpha}$ | 19 (5-25) | 15 (5-36) | N.S. |
| $6\text{-}kPGF_{1\alpha}$ | 16 (8-30) | 13 (7-24) | N.S. |
| $TxB_2$ | 71 (1-141) | 42 (3-149) | N.S. |
| $LTC_4$ | 16 (6-53) | 9 (1-17) | 0.01 |
| $LTB_4$ | 75 (15-138) | 96 (23-279) | N.S. |

Medians with range. $CS^-$ and $CS^+$ = asthma groups treated without and with inhaled corticosteroids, respectively.

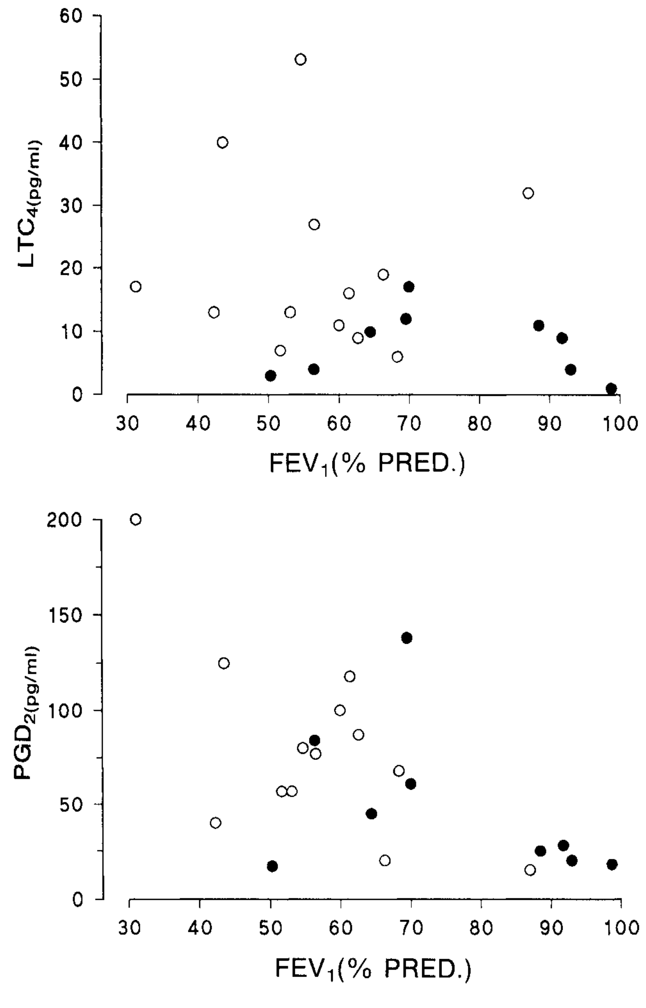

FIG. 1. Relation of BAL $LTC_4$ and $PGD_2$ levels on $FEV_1$ % predicted in asthmatic subjects treated with inhaled corticosteroids (closed circles) and asthmatic subjects treated with inhaled $\beta_2$-agonists alone (open circles). For details, see text.

*Correlation with lung function:* $LTC_4$ levels correlated significantly with $FEV_1$ % pred. (rho = −0.46, $p = 0.03$) (Fig. 1). $PGD_2$ levels correlated significantly with $FEV_1$ % pred. (rho = −0.62, $p = 0.002$), as did $PC_{20}$ histamine (rho = −0.50, $p = 0.02$) and the reversibility of airways obstruction (rho = −0.52, $p = 0.01$).

### Discussion

This study started from the hypothesis that suppression of inflammatory processes in the airways underlies the improvement in lung function observed after long-term treatment with inhaled corticosteroids. AA metabolite levels are considered as biochemical markers of on-going chronic airway inflammation in the airways of asthmatic subjects.[3–5] Therefore, we investigated whether differences in BAL AA metabolite levels could be detected between subjects treated with $\beta_2$-agonists and inhaled corticosteroids for 2.5 years and those treated with inhaled $\beta_2$-agonists alone. Results from this study show that the BAL $LTC_4$ levels are significantly lower in asthmatic patients treated with inhaled corticosteroids. The same trend is observed for $PGD_2$ levels. The median BAL AA metabolite values of the corticosteroid treated group were within the same level as those from a control group of eight non-smoking, non-atopic healthy subjects who were concurrently analysed during the same procedure.[10]

This is the first study in which AA metabolite levels were measured after long-term treatment with inhaled corticosteroids. In contrast to our results, short-term treatment with prednisolone 60 mg/day has been reported to have no significant effect on BAL fluid AA metabolite levels in asthmatic subjects, although the *in vitro* synthesis of AA metabolites by BAL cells was decreased.[11] A lower production of $LTC_4$ by BAL cells was also found in asthmatic subjects who had been treated for more than 2 years with 5–15 mg prednisone,[12] which is in line with our results. A role for cysteinyl leukotrienes in the pathophysiology of asthma is suggested by findings that leukotrienes induce airway obstruction, increase airway hyper-responsiveness and increase mucous secretion.[3] After oral treatment with a leukotriene $D_4$ receptor antagonist a significant reduction in asthma symptoms and improvement of lung function has been reported in asthmatic subjects.[13] In the light of current knowledge, several mechanisms can be postulated to explain the reduced $LTC_4$ levels in the CS-treated group. First, corticosteroids exert a decreased cellular AA metabolite synthesis by inhibiting phospholipase $A_2$ activation through the generation of lipocortin.[14] Results from this study suggest that cells that predominantly release $LTC_4$ (eosinophils, alveolar macrophages, mast cells) are more sensitive to corticosteroid treatment. Secondly, corticosteroids may selectively inhibit the transcription of cytokines from airway cells[6,15,16] that may regulate $LTC_4$ release. IL-3, IL-5 and GM-CSF have been shown to prime human basophils, eosinophils and neutrophils for augmented release of $LTC_4$ after stimulation by a second agonist.[17–19]

Results of this study suggest an effect of long-term corticosteroid treatment on BAL $PGD_2$ levels as well. $PGD_2$ is a potent airway constrictor and is implicated in the increase in airway responsiveness.[3] $PGD_2$ is synthesized by a variety of airway cells. It has been observed that the *in vitro* $PGD_2$ synthesis by human lung mast cells was not affected by glucocorticoids.[20] The $PGD_2$ synthesis by human alveolar macrophages upon stimulation by calcium ionophore A23187 was, however, significantly inhibited by methyl prednisolone.[21] If *in vitro* results can be extrapolated to the *in vivo* situation, the results favour a role for $PGD_2$ release by alveolar macrophages in the chronic inflammatory process in asthma.

We did not find a difference in total or differential cell numbers between the groups. Previous findings in studies on the effect of short-term corticosteroid treatment (6 weeks to 4 months) on BAL cell numbers are not consistent, although in the majority a reduction of BAL eosinophil numbers was found.[15,16,22,23] It cannot be excluded that we did not find differences as a consequence of group sizes that were too small. Inflammatory processes in the airways are, however, probably better reflected by cell activation than increased cell numbers in the BAL fluid, as has been found in this study.

In conclusion, this study shows that BAL $LTC_4$ levels of asthmatic subjects were significantly lower after 2.5 years inhaled corticosteroid therapy. The results suggest that corticosteroids may exert their beneficial effect on lung function via a mechanism in which inhibition of $LTC_4$ synthesis in the airways is involved.

### References

1. Kerstjens HAM, Brand PLP, Hughes MD, *et al.* A comparison of bronchodilator therapy with or without inhaled corticosteroid therapy for obstructive airways disease. *N Engl J Med* 1992; **327:** 1413–1419.
2. Haahtela T, Järvinen M, Kava T, *et al.* Comparison of a $\beta_2$-agonist, terbutaline, with an inhaled corticosteroid, budesonide, in newly detected asthma. *N Engl J Med* 1991; **325:** 388–392.
3. Arm JP, Lee TH. The pathobiology of bronchial asthma. *Advances in Immunology* 1992; **51:** 323–382.
4. Wenzel SE, Larsen GL, Jonston K, Voelkel NF, Westcott JY. Elevated levels of leukotriene $C_4$ in bronchoalveolar lavage fluid from atopic asthmatics after endobronchial allergen challenge. *Am Rev Respir Dis* 1990; **142:** 112–119.
5. Liu MC, Hubbard WC, Proud D, *et al.* Immediate and late inflammatory responses to ragweed antigen challenge of the peripheral airways in allergic asthmatics. Cellular, mediator, and permeability changes. *Am Rev Respir Dis* 1991; **144:** 51–58.
6. Barnes PJ, Pedersen S. Efficacy and safety of inhaled corticosteroids in asthma. *Am Rev Respir Dis* 1993; **148:** S1–S26.
7. Rijcken B, Schouten JP, Weiss ST, Speizer FE, Van der Lende R. The relationship of nonspecific bronchial responsiveness to respiratory symptoms in a random population sample. *Am Rev Respir Dis* 1987; **136:** 62–68.
8. Summary and recommendations of a workshop on the investigative use of fiberoptic bronchoscopy and bronchoalveolar lavage in asthmatics. *Am Rev Respir Dis* 1985; **132:** 180–182.
9. Zijlstra FJ, Vincent JE, Mol WM, Hoogsteden HC, Van Hal PW, Jongejan RC. Eicosanoid levels in bronchoalveolar lavage fluid of young female smokers and non-smokers. *Eur J Clin Invest* 1992; **22:** 301–306.
10. Oosterhoff Y, Kauffman HF, Rutgers B, Zijlstra FJ, Koëter GH, Postma DS. Inflammatory cell number and mediators in bronchoalveolar lavage fluid and peripheral blood in subjects with asthma with increased nocturnal airway narrowing. *J Allergy Clin Immunol* 1995; **96**: 219–229.
11. Dworski R, Fitzgerald GA, Oates JA, Sheller JR, Workman R, Prakash C. Effect of oral prednisone on airway inflammatory mediators in atopic asthma. *Am J Respir Crit Care Med* 1994; **149:** 953–959.
12. Tanikazi Y, Kitani H, Okazaki M, Mifune T, Mitsunobu F, Kimura I. Changes in the proportions of bronchoalveolar lymphocytes, neutrophils and basophillic cells and the release of histamine and leukotrienes from bronchoalveolar cells in patients with steroid-dependent intractable asthma. *Int Arch Allergy Immunol* 1993; **101:** 196–202.
13. Spector SL, Smith LJ, Glass M, and the ACCOLATE asthma trialists group. Effects of 6 weeks of therapy with oral doses of ICI 204,219, a leukotriene $D_4$ receptor antagonist, in subjects with bronchial asthma. *Am J Respir Crit Care Med* 1994; **150:** 618–623.
14. Goulding NJ, Godolphin JL, Sharland PR, *et al.* Anti-inflammatory lipocortin-1 production by peripheral leucocytes in response to hydrocortisone. *Lancet* 1990; **335:** 1416–1418.
15. Robinson D, Hamid Q, Ying S, *et al.* Prednisolone treatment in asthma is associated with modulation of bronchoalveolar lavage cell interleukin-4, interleukin-5, and interferon-τ cytokine gene expression. *Am Rev Respir Dis* 1993; **148:** 401–406.
16. Trigg CJ, Manolitsas ND, Wang J, *et al.* Placebo-controlled immunopathologic study of four months of inhaled corticosteroids in asthma. *Am J Respir Crit Care Med* 1994; **150:** 17–22.
17. Bischoff SC, Brunner T, de Weck AL, *et al.* Interleukin 5 modifies histamine release and leukotriene generation by human basophils in response to diverse agonists. *J Exp Med* 1990; **172:** 1577–1582.
18. Takafuji S, Bischoff SC, de Weck AL, *et al.* Interleukin 3 and interleukin 5 prime human eosinophils to produce leukotriene $C_4$ in response to soluble agonists. *J Immunol* 1991; **147:** 3855.
19. Dahinden CA, Zingg J, Maly FE, *et al.* Leukotriene production in human neutrophils primed by recombinant human granulocyte/macrophage colony-stimulating factor and stimulated with the complement component C5a and FMLP as second signals. *J Exp Med* 1988; **167:** 1281.
20. Schleimer RP, Schulman ES, MacGlashan DW, *et al.* Effects of dexamethasone on mediator release from human lung fragments and purified human lung mast cells. *J Clin Invest* 1983; **71:** 1830–1835.
21. Balter MS, Eschenbacher WL, Peters-Golden M. Arachidonic acid metabolism in cultured alveolar macrophages from normal, atopic, and asthmatic subjects. *Am Rev Respir Dis* 1988; **138:** 1134–1142.
22. Duddridge M, Ward C, Hendrick DJ, Walters EH. Changes in bronchoalveolar lavage inflammatory cells in asthmatic patients treated with high dose inhaled beclomethasone dipropionate. *Eur Respir J* 1993; **6:** 489–497.
23. Wilson JW, Djukanovic R, Howarth PH, Holgate ST. Inhaled beclomethasone dipropionate downregulates airway lymphocyte activation in atopic asthma. *J Respir Crit Care Med* 1994; **149:** 86–90.

**Received 6 July 1995;**
**accepted in revised form 14 September 1995**
